# Grade 1 and 2 Chondrosarcomas of the Chest Wall: CT Imaging Features and Review of the Literature

**DOI:** 10.3390/diagnostics12020292

**Published:** 2022-01-24

**Authors:** Filippo Del Grande, Shivani Ahlawat, Edward McCarthy, Laura M. Fayad

**Affiliations:** 1 The Russel H. Morgan Department of Radiology and Radiological Science, Johns Hopkins University, Baltimore, MD 21205, USA; sahlawa1@jhmi.edu (S.A.); lfayad1@jhmi.edu (L.M.F.); 2 Clinica di Radiologia EOC, Via Tesserete, 6900 Lugano, Switzerland; 3 Department of Pathology, Johns Hopkins University, Baltimore, MD 21205, USA; mccarthy@jhmi.edu

**Keywords:** chondrosarcoma, chest wall tumor, bone tumor, CT scan

## Abstract

The purpose of our retrospective article is to review the CT imaging features of chondrosarcomas of the chest wall with pathologic correlation. For 26 subjects with biopsy-proven chondrosarcomas of the chest wall, two musculoskeletal radiologists retrospectively reviewed 26 CT scans in consensus. Descriptive statistics were performed. The mean tumor size was 57 mm. Twenty (20/26, 77%) chondrosarcomas were located in the ribs and six (6/26, 23%) in the sternum. The majority were lytic (19/26, 73%) with <25% calcification (15/26, 58%), and with a soft tissue mass (22/27, 85%). In this study CT features of grade 1 chondrosarcoma overlapped with those of grade 2 tumors. In conclusion, chondrosarcomas of the chest wall are generally lytic with an associated soft tissue mass, showing little calcified matrix and low-to-intermediate grade.

## 1. Introduction

Chondrosarcomas (CS) of the chest wall are rare lesions, but they represent the most common primary malignant bone tumors of the chest wall [1,2]. Both primary [3,4,5,6,7,8,9,10] and secondary forms of CS [11,12,13] have previously been reported. 

The imaging features of CS of the chest wall have been insufficiently described, with limited reports depicting the imaging features for radiography [6,7,9], cross-sectional imaging [2,4,6,7,8,9,12,14], and PET [4,15,16,17]. There are, however, several reports in the surgical literature regarding the clinical features and prognosis of CS of the chest wall [5,8,13,18,19,20]. It is important for the imaging interpreter to consider CS in the differential diagnosis of a chest wall mass, especially due to the knowledge that some histologic features of CS can overlap with enchondroma, as has already been described in the extremities [21]. 

While radiography of a chest wall mass is often the first-line imaging modality for tumor detection, it is often inadequate for characterization. Therefore, cross-sectional imaging techniques are important modalities for the detection and characterization of CS of the chest wall. The purpose of our study was to retrospectively review the CT imaging features of chondrosarcomas (CS) of the chest wall with pathologic correlation.

## 2. Materials and Methods

### 2.1. Patients

The study was approved by our institutional review board (IRB). Informed consent was waived. Details of subjects who had a surgical specimen with a diagnosis of chest wall CS at our institution from 1988 to 2016 were retrieved from a pathology database. Inclusion criteria were patients with a histologically proven chest wall CS, cross-sectional imaging with CT exam and no neoadjuvant therapy prior to their imaging studies. Exclusion criteria were patients without histology or CT imaging documentation, and those with imaging that was performed following surgery or other treatments.

### 2.2. Image Acquisition

CT protocols were heterogenous, depending on the institution where the exams had been performed. All CT exams included axial CT, and 16/26 (62%) patients had sagittal and coronal reconstructions. 

### 2.3. Image Analysis

Two musculoskeletal radiologists (SA and FDG) with 7 and 17 years of experience in musculoskeletal imaging interpretation, respectively, reviewed the CT imaging features of 26 biopsy-proven chondrosarcomas (CS) of the chest wall in consensus and blinded to clinical information. 

The size, location, character (lytic, with chondroid matrix calcification or mixed), calcification percentage (none, 1–25%, 26–50%, 51–75%, >75% lesion calcification), location (central or peripheral, related to the bone), as well as the presence or absence of a soft tissue mass or fracture were recorded. Lesions growing outside the bone with soft tissue Hounsfield units were considered soft tissue masses. 

### 2.4. Pathologic Interpretation 

One pathologist (EM) with more than 40 years’ experience in bone tumors reviewed all specimens and graded tumors as grade 1, 2, or 3 based on the following scheme: Grade 1 chondrosarcomas showed minimal cellular atypia with occasional binucleated cells; rarely, a few cells had slightly larger nuclei and more cytoplasm. Abundant extracellular matrix was present and there was no evidence of myxoid change or mitotic activity. Grade 2 chondrosarcomas had larger and pale nuclei with visible chromatin. There was mild nuclear polymorphism and the cytoplasm was more abundant. Mitotic figures were extremely rare. Grade 2 lesions were slightly more cellular and, rarely, myxoid change was present. Grade 3 chondrosarcomas had significant cellularity with pleomorphic nuclei. There was often extensive myxoid change with abundant spindle-shaped tumor cells. Mitotic figures were present at the rate of 2 to 10 per high-power field, and, focally, the exterior cellular matrix was sparse. Biopsy material was available, either from surgical excision or percutaneous sampling. In nineteen out of twenty-six (73%) cases, samples from surgical biopsies were available, and in 7/26 (27%) cases, samples from percutaneous core needle biopsies were available. All imaging was available during pathological interpretation.

### 2.5. Statistical Analysis

Descriptive statistics were tabulated manually in an Excel file for demographics (age with mean and range, and gender), location and size of the lesion, as well as imaging features and histologic grades as detailed above. The Mann–Whitney U test and Fisher’s exact test were used to perform comparisons between grade 1 and 2 CS using STATA17 (StataCorp., College Station, TX, USA). Statistical significance was set at 5% (*p* < 0.05).

## 3. Results

We retrieved reports of 54 subjects with chest wall CS. Twenty-eight subjects (28/54, 52%) were excluded due to the lack of a complete CT. According to our inclusion criteria, we identified 26 (26/54, 48%) patients with CT images and chest wall CS (17/26 males, 9/26 females), with a median age of 61 years (age range: 25–88 years old). A histologic analysis revealed 10 grade 1 CS (10/26, 38%), 16 grade 2 CS (16/26, 62%), and no grade 3 lesions. 

No statistically significant differences were detected between grade 1 and 2 CS. 

Table 1 summarizes the demographics and general tumor characteristics separated by grade.

**Figure 1:**
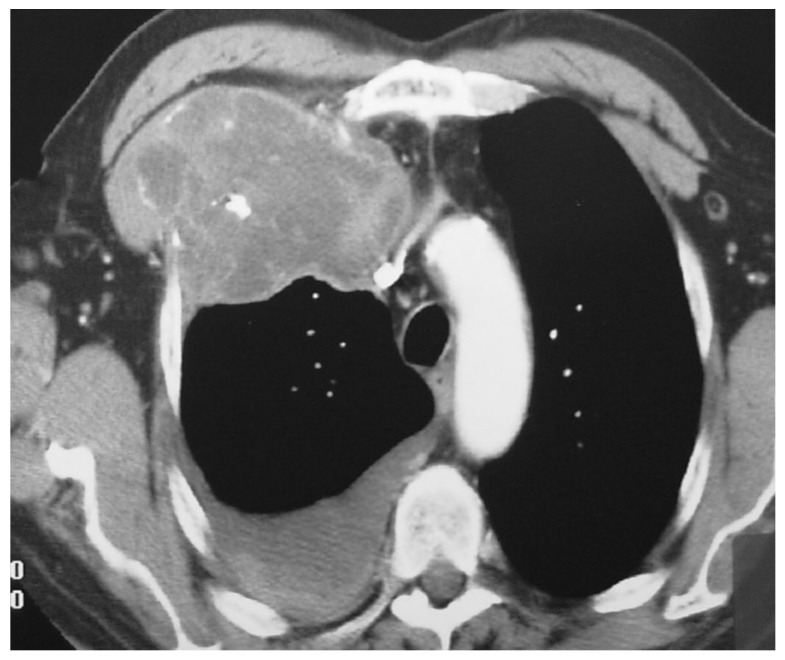
A 72-year-old male patient with grade 2 costochondral junction chondrosarcoma. Contrast-enhanced axial chest CT shows a large (90 mm) soft tissue mass at the right costochondral junction with small calcifications, necrotic areas, mediastinal invasion and right pleural effusion.

The mean tumor size was 57 mm (range: 20–151 mm) (Figure 1).

**Figure 2:**
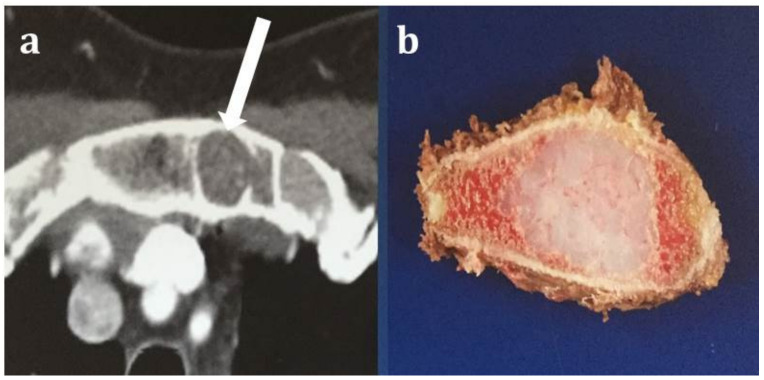
A 49-year-old female patient with grade 2 chondrosarcoma (long white arrow) of the sternum. Contrast-enhancement axial CT with soft tissue window (**a**) shows a lytic lesion with a few punctate calcifications. Gross pathology specimen resection of the sternum (**b**) showing a white-yellow tumor surrounded by bone marrow. Based on imaging, it is not possible to differentiate the lesion from an enchondroma. Enchondromas in the sternum are extremely rare and a solid lesion in the sternum should raise concerns for malignancy until proven otherwise.

**Figure 3:**
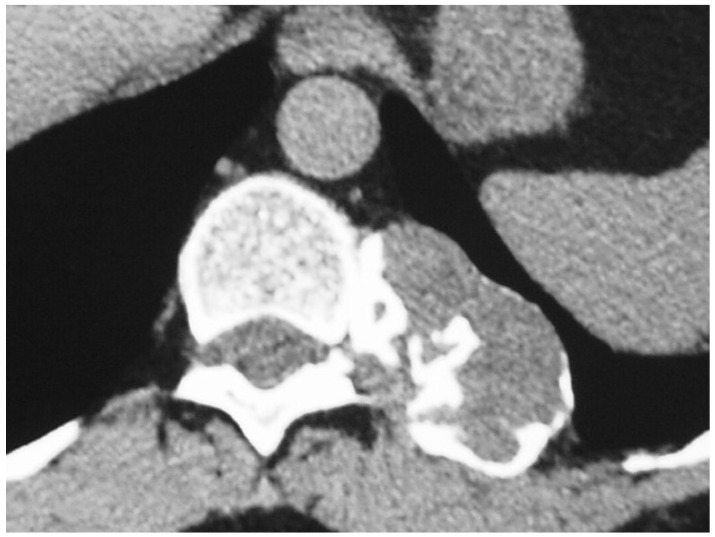
A 62-year-old female patient with grade 2 chondrosarcoma at the posterior rib adjacent to the costovertebral junction. Axial chest CT shows a soft tissue mass at the posterior rib with calcifications.

Nineteen lesions were located at the costochondral junction (19/26, 73%), six lesions were located in the sternum (6/26, 23%) (Figure 2), and one lesion (1/26, 4%) was located in the posterior rib adjacent to the costovertebral joint (Figure 3).

**Figure 4:**
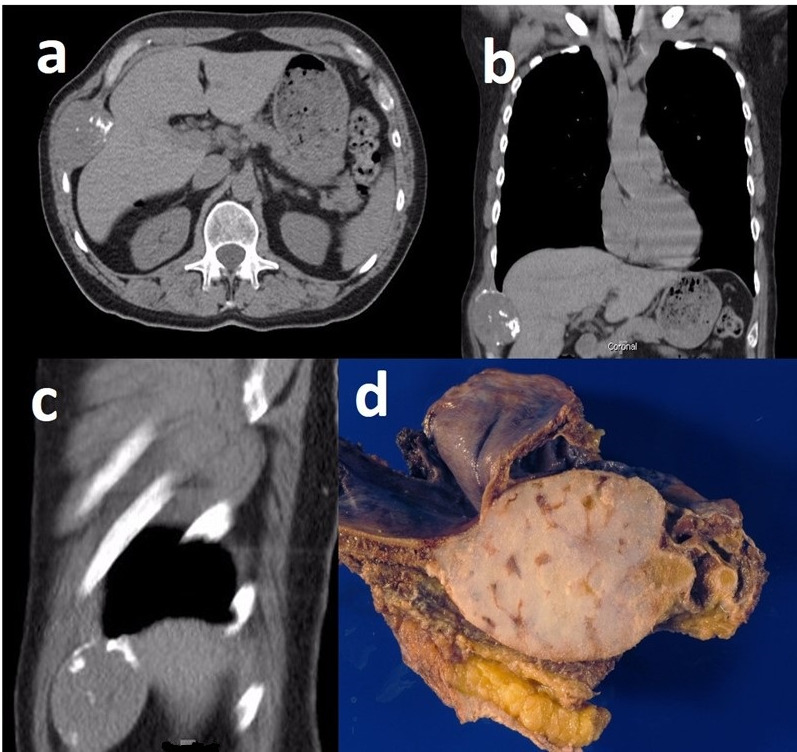
A 74-year-old male patient with grade 1 costochondral junction chondrosarcoma. Axial chest CT (**a**), and coronal (**b**) and sagittal (**c**) multi-planar reconstruction (MPR) CT show a large soft tissue mass at the costochondral junction. Gross pathology specimen (**d**) resection of the rib with chondrosarcoma.

The typical appearance of CS was lytic (16/26, 62%). More than half of the subjects (15/26, 58%) showed less than 25% calcifications, and only one subject (1/26, 4%) had more than 75% calcifications in the lesion. Commonly, CS were associated with a soft tissue mass (22/26, 85%) (Figure 4), and only two CS (2/26, 8%) had pathological fractures. 

Fourteen CS (14/26, 54%) (13 located at the costochondral junction and one on the posterior rib) were considered to be peripherally located, whereas 12 CS (12/26, 46%) were considered to be centrally located (6 located in the sternum and 6 at the costochondral junction). The six CS located in the sternum were all centrally located. Out of 19 CS of the osteochondral junction, 14 (74%) were peripherally located. 

The vast majority of the lesions (4/5, 80%) showed less than 25% heterogeneity after contrast medium injection. None of the CS showed evidence of perilesional bone marrow edema, perilesional enhancement, and/or perilesional soft tissue edema. Table 2 shows the CT imaging features separated by the grade of the tumor.

## 4. Discussion

In our retrospective series, we found that grade 1 and grade 2 chest wall CS are typically lytic lesions located at the costochondral junction, with an associated soft tissue mass, and with little mineralization. Grade 1 and 2 CS were not statistically different for tumor location in the chest wall, tumor size, tumor location within the bone, presence or absence of chondroid matrix, fractures and/or soft tissue masses. 

Compared with prior reports in the literature, our findings are corroborated by Al-Refaie et al., who reported the demographics and treatment of 45 chest wall CS, without a comprehensive assessment of the imaging. The authors found that CS were slightly more common in male than female patients, and were mostly located at the costochondral junction, similarly to our investigation. Histologically, the majority of CS were low-grade lesions, with a minority of intermediate-grade lesions, and no high-grade lesions were reported in the latter study [18].

Cross-sectional imaging features of chest wall CS have only been insufficiently described [2,4,6,7,8,9,12,14]. Some cross-sectional imaging features that we observed are similar to the imaging features described in chondrosarcomas elsewhere in the body, and, as such, were expected. In particular, as commonly observed in chondroid lesions, the majority of the lesions showed a low-to-isointense signal on spin-echo T1-weighted sequences and a high signal intensity on fluid-sensitive sequences. Following contrast enhancement, a ring-like pattern was more common than a solid pattern of enhancement. 

Several features of CS of the chest wall observed in our study were different from those that have been reported in the aicular skeleton and other sites of the axial skeleton. Unlike CS of the pelvic bones and extremities, in which calcification is common [22,23], in CS of the chest wall, calcification is much less common. In one prior investigation, almost every CS of the aicular skeleton showed more than one third calcified matrix, but the chest wall CS in our series showed surprisingly less calcification, with the majority of the patients having less than 25% of their lesions containing calcification [22]. We can speculate that the difference in calcification pattern between extremity CS and chest wall CS is probably attributed to the overall relatively low percentage of calcification in large chest wall soft tissue masses. 

Interestingly, the CT imaging features were similar between low-grade and intermediate-grade lesions. In other words, due to our small series and limited imaging availability, the lesion appearance, percentage of calcification, presence of soft tissue mass, and size of the lesions could not be used to draw any conclusions regarding tumor grade. Furthermore, in some previous reports, the pattern and the degree of contrast medium enhancement did not discriminate between CS and enchondroma [22,24].

In order to avoid unnecessary biopsies or inadequate treatments, it is crucial to identify imaging features in order to discriminate between enchondroma and low-grade CS throughout the body. Similarly to CS, enchondroma are usually located in the rib or at the costochondral junction [2,25], but enchondromas of the sternum are very rare, with only isolated reports [26,27,28,29]. In the chest wall, enchondromas generally appear as small (typically less than 3.5 cm), well-defined lytic lesions [30], while we showed that CS of the chest wall are generally associated with more aggressive features, including a soft tissue mass. In addition, demographic and clinical information has been reported to be useful for differentiating between enchondromas and CS elsewhere in the body, including older patient age, male gender, and pain [22]. It is also worth noting that pathologists cannot always differentiate enchondroma from low-grade chondrosarcoma by cytology and histology, further accentuating the importance of imaging features, and the integration of imaging studies and clinical information with a pathologic diagnosis [21]. Therefore, in the management of chondroid lesions, a percutaneous biopsy is not routinely performed in many centers, as it is prone to sampling error and the final histologic diagnosis is strongly reliant on the imaging features.

**Figure 5:**
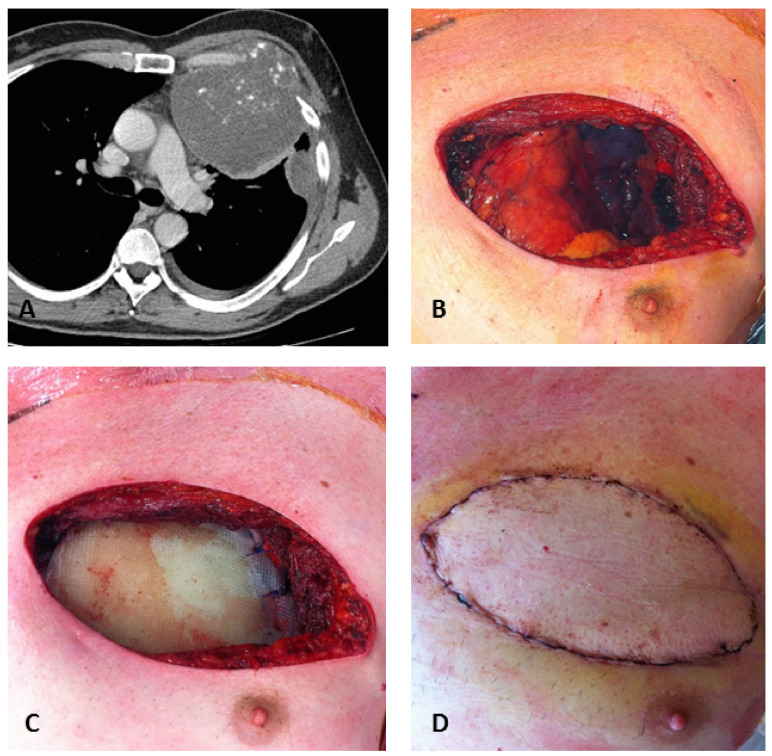
A 67-year-old male patient with a high-grade chondrosarcoma of the costochondral junction. Axial contrast-enhanced CT image (**A**) shows a large chondrosarcoma of the left costochondral junction with less than 25% calcified matrix and a large soft tissue mass. Little left pleural effusion is also present. Intraoperative views show radical resection (**B**), reconstruction by composite rigid prosthesis (**C**), and latissimus dorsi flap reconstruction (**D**). Courtesy of Prof Francesco Petrella, Istituto Europeo di Oncologia (IEO), Milan, Italy.

**Figure 6:**
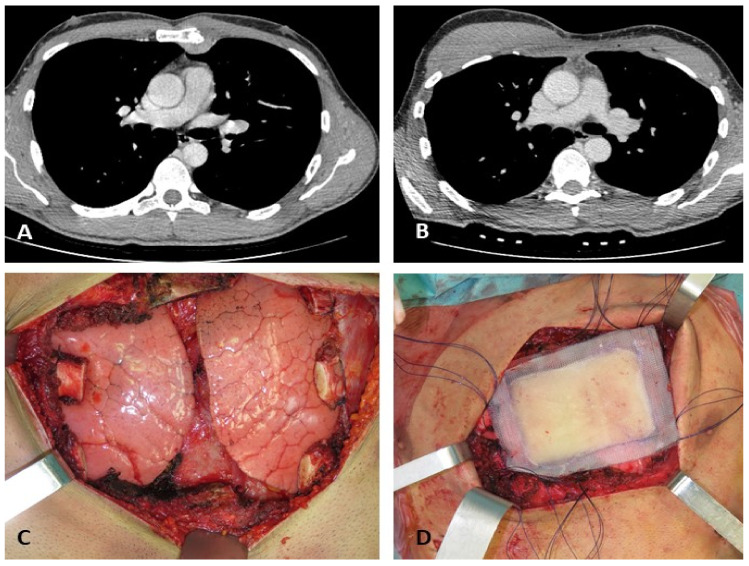
A 35-year-old male patient with a low-grade chondrosarcoma of the sternum. Chondrosarcoma of the sternum with pre- and post-radical resection axial contrast-enhanced CT images (**A**,**B**). Intraoperative views (**C**,**D**) show radical resection and reconstruction by composite rigid prosthesis. Courtesy of Prof Francesco Petrella, Istituto Europeo di Oncologia (IEO), Milan, Italy.

A correct diagnosis is paramount in starting a correct therapeutic approach, in decreasing the recurrence rate, and in increasing patient survival. Although chest radiography is typically the first-line study of evaluation for osseous lesions, the location of chest wall CS requires cross-sectional imaging for the characterization and definition of tumor extent. CT imaging of the chest is, therefore, the next modality of investigation, as CT offers information for the characterization of the tumor as chondroid. Subsequently, MRI is useful for defining additional aggressive features (such as perilesional edema), as well as the full extent of the tumor within the bone marrow for optimal treatment planning and identification of the margins. It should be noted that CS of the chest wall are relatively resistant to chemotherapy and radiotherapy, and, as such, a radical resection with wide margins is favored [31] (Figure 5 and Figure 6).

Following adequate wide-margin resection, patients have been reported to have a 10-year recurrence rate of 7–17%, whereas patients with a local excision only had a reported recurrence rate of 50–57%. Similarly, ten-year survival rates of 96–100% and 65–68% have been reported for patients treated with wide resection and with local excision, respectively [32,33].

Our study has some limitations. Firstly, our sample size is relatively small, but chest wall CS are rare lesions, even in a tertiary referral center. In addition, understanding the proportion of chest wall lesions present with similar features to those we have described as features of chest wall CS would be valuable information, although our study design does not allow exploration of this topic. Secondly, the imaging protocols were heterogeneous, due to the long time span over which the cases were retrieved. Thirdly, clinical presentation, such as the presence of pain, was available for only a small portion of the patients, and was, therefore, not reported.

## 5. Conclusions

In conclusion, chest wall CS are usually lytic lesions with associated soft tissue masses, and have little calcification. The location, lesion size, calcification size and percentage, associated soft tissue mass, and peripheral or central location within the bone are similar for grade 1 and grade 2 CS.

## Figures and Tables

**Table 1:** Demographics separated by grade.

Imaging Feature	Variables	Grade 1 11/26 (42%)	Grade 2 15/26 (58%)	*p*-Value	All CS 26/26 (100%)
Age	Mean and range in years	60 (33–84)	62 (25–88)	0.788	61 (25–88)
Gender	Males	9/11 (82%)	8/15 (53%)	0.217	17/26 (65%)
	Females	2/11 (18%)	7/15 (47%)		9/26 (35%)

**Table 2:** CT imaging features of CS, including separation by grade.

Imaging Features	Variables	Grade 1 11/26 (42%)	Grade2 15/26 (58%)	*p*-Value	All CS 26/26 (100%)
Tumor location	Costochondral junction	9/11 (82%)	10/15 (67%)	0.804	19/26 (73%)
	Sternum	2/11 (18%)	4/15 (27%)		6/26 (23%)
	Posterior rib	0/11 (0%)	1/15 (6%)		1/26 (4%)
Tumor size	Mean in mm (range)	56 (31–151)	57 (20–127)	0.435	57 (20–151)
	Median in mm (range)	40 (31–151)	46 (20–127)		45 (20–151)
Character	Lytic only	0/11 (0%)	3/15 (20%)	0.238	3/26 (12%)
	mixed	11/11 (100%)	12/15 (80%)		23/26 (88%)
	chondroid matrix only	0/11 (0%)	0/15 (0%)		0/26 (0%)
Chondroid Matrix	0–25%	5/11 (45%)	10/15 (67%)	0.551	15/26 (58%)
	25–50%	4/11(36%)	3/15 (20%)		7/26 (27%)
	50–75%	1/11 (9%)	2/15 (13%)		3/26 (12%)
	75–100%	1/11 (9%)	0/15 (0%)		1/26 (4%)
Fracture	No	9/ 11(82%)	15/15(100%)	0.169	24/26 (92%)
	Yes	2/11 (18%)	0/15 (0%)		2/26 (8%)
Tumor location within the bone	Central	5/11 (45%)	7/15 (47%)	1.000	12/26 (48%)
	peripheral	6/11 (55%)	8/15 (53%)		14/26 (52%)
Soft tissue mass	No	3/11(27%)	1/15 (7%)	0.279	4/26 (15%)
	Yes	8/11(73%)	14/15 (93%)		22/26 (85%)
